# Editorial for the Special Issue on Micro/Nano-Chip Electrokinetics, Volume III

**DOI:** 10.3390/mi11050482

**Published:** 2020-05-08

**Authors:** Shizhi Qian, Xiangchun Xuan

**Affiliations:** 1 Department of Mechanical and Aerospace Engineering, Old Dominion University, Norfolk, VA 23529, USA; 2 Department of Mechanical Engineering, Clemson University, Clemson, SC 29634, USA; xcxuan@clemson.edu

With the support from contributors and the help from peer reviewers, the Special Issue on Micro/Nano-Chip Electrokinetics (Volume III) published fourteen regular research articles and one review article. Based on the involved electrokinetic phenomena, these papers can be classified into the following six groups as summarized below.

(1) **Organs-on-a-chip (1 paper).** The review on organs-on-a-chip [1] reviewed the principles, fabrication techniques, and recent progress of organs-on-chip, which aims to achieve a complete functionality including the inclusion of specific conditions for the organ or tissue such as pressure, flow rate, pH, osmotic pressure, nutrient content, toxins presence, among other properties. Electrokinetic phenomena, such as electroosmotic pumps, can be applied to the organs-on-chip applications.
(2) **Newtonian Electroosmotic Flow (EOF) (3 papers).** EOF has been widely used to pump fluids in micro/nanofluidic applications. Khan and Dutta [2] derived an analytical solution of time-periodic EOF through a microtube with heterogeneous distribution of zeta potential. Ye et al. [3] developed a low-voltage high flow rate 3D EOF pump, which achieved a flow rate of 5.69 nL/min at a driving voltage of 2 V. Li et al. [4] numerically simulated EOF and ionic mass transport in a microchannel with an ion exchange membrane (IEM), and investigated the performance of seawater desalination of the system. Newtonian fluid was considered in these studies.
(3) **Non-Newtonian Electroosmotic Flow (EOF) (3 papers).** Choi et al. [5] derived an analytical solution of EOF of power-law fluid in a slit microchannel with different zeta potentials at the top and bottom walls. Chen et al. [6] simulated EOF of viscoelastic Linear Phan–Thien–Tanner (LPTT) fluid in a microchannel under various conditions, and found out that EOF of viscoelastic fluid was higher than that of Newtonian fluid under the same conditions. Mei and Qian [7] also numerically simulated EOF of LPTT fluid through a nano-slit connecting two reservoirs on both sides, and significant enhancements of both flow rate and ionic conductance were observed for viscoelastic fluid compared to Newtonian fluid.
(4) **Induced-Charge Electroosmosis (ICEO) (3 papers).** Du et al. [8] developed a unique concept of multifrequency induced-charge electroosmosis (MICEO) on ideally polarizable surfaces of a series of parallelly-placed metal strips. The proposed MICEO combines the transverse AC electroosmotic vortex flow and the axial traveling-wave electroosmotic pump motion under external dual-Fourier-mode AC electric fields. Jiang et al. [9] used ICEO in 3D composite electrode layouts to concentrate particles. Du et al. [10] utilized the ICEO flow controlled by AC field-effect transistor to generate secondary flow for mixing enhancement.
(5) **Electrohydrodynamics (EHD) (2 papers)**. Qian et al. [11] proposed a simple and easily implemented method for achieving tunable-focus liquid lenses. By corona discharge in the air, electro-pressure with a magnitude of 10 Pa was generated at the interface between liquid silicone and air, and the resulting electro-pressure was utilized to tune liquid-lens. Liu and Liu [12] numerically investigated the EHD phenomena of sessile droplets on hydrophobic surfaces under non-uniform electric fields using the phase field method. They analyzed the dynamic behaviors of the electro-driven deformation and motion of water droplets in the oil phase.
(6) **Dielectrophoresis (DEP) (3 papers)**. Islam et al. [13] characterized the DEP response of *Candida albicans*, *Candida tropicalis* and *Candida parapsilosis* using 3D carbon microelectrodes. Peña et al. [14] demonstrated the first time use of insulator-based dielectrophoresis (iDEP) to study bacteriophages, possibly the most abundant and genetically diverse biological entities on earth. Yin et al. [15] integrated DEP and microstructure filtration to achieve multi-stage particle and cell separation.

We appreciate the contributors who submitted their articles to this Special Issue. We would like to thank many reviewers for taking time and effort to review manuscripts submitted to this Special Issue. We also acknowledge many assistant editors from Micromachines Editorial Office, and we could not have the third volume without their help and support. The first, second, and third volumes of the Special Issue on Micro/Nano-Chip Electrokinetics can be accessed through the following links:

**Volume I**

https://www.mdpi.com/journal/micromachines/special_issues/micro_nano_chip_electrokinetics

**Volume II**

https://www.mdpi.com/journal/micromachines/special_issues/micro_nano_chip_electrokinetics_v2

**Volume III**

https://www.mdpi.com/journal/micromachines/special_issues/micro_nano_chip_electrokinetics_v3
